# Young People's Voices in Developing Messages on Brain Health: An Initiative to Promote Brain‐Protective Lifestyle Habits Among Young Adults

**DOI:** 10.1111/hex.70926

**Published:** 2026-10-25

**Authors:** Soraya Moradi‐Bachiller, David Bartrés‐Faz, Holger Fröhlich, Jean Georges, Dianne Gove, Jesús Rodrigo, Jussi Tohka, Ana Díaz‐Ponce

**Affiliations:** ^1^ Alzheimer Europe Luxembourg; ^2^ Departament de Medicina, Facultat de Medicina i Ciències de la Salut Universitat de Barcelona, and Institut de Neurociències, Universitat de Barcelona, Institut d'Investigacions Biomèdiques August Pi i Sunyer (IDIBAPS) Barcelona Spain; ^3^ Department of Biomedical AI & Data Science Fraunhofer Institute for Algorithms and Scientific Computing (SCAI), Schloss Birlinghoven Sankt Augustin Germany; ^4^ University Hospital Bonn, Institute for Digital Medicine, University of Bonn Bonn Germany; ^5^ Bonn‐Aachen International Center for IT (b‐it) University of Bonn Bonn Germany; ^6^ Confederación Española de Alzheimer y otras demencias, Pamplona Navarra Spain; ^7^ A.I. Virtanen Institute for Molecular Sciences University of Eastern Finland Kuopio Finland

**Keywords:** brain health, dementia, emerging adulthood, public health, Public Involvement, young adults

## Abstract

**Introduction:**

The work presented in this article was conducted as part of two European‐funded projects (ADIS and Pattern‐Cog) examining the topics of dementia risk reduction and timely diagnosis. Health messages promoting and educating people about brain health (defined by the World Health Organisation as the state of brain functioning across cognitive, sensory, social‐emotional, behavioural and motor domains, allowing a person to realise their full potential over the life course, irrespective of the presence or absence of disorders) are beginning to appear in the public domain. However, to the best of our knowledge, there are no campaigns specifically developed to inform, educate and promote brain health among young adults. This seems counterintuitive given the potential for prevention, particularly at this important transitional phase in a young person's life. This article reports feedback collected during consecutive four online and face‐to‐face Public Involvement (PI) consultations with nine young adults from Spain that focused on understanding their perspectives on dementia and brain health promotion messages. A subsequent aim was to describe how this informed the development of a brain health campaign.

**Approach:**

Notes from the consultations were summarised and organised using thematic analysis techniques. Meeting summaries informed the development of a brain health campaign targeting young adults in Spain—‘Yo Me Celebro'. The campaign was developed in a partnership approach with the young adults.

**Outcomes:**

Four main topics related to brain health were summarised from the PI consultations: (1) understanding, (2) terminology, (3) barriers and limitations to change and (4) preferred approach. Many learning points and limitations were identified from these topics (i.e., framing brain health in a positive, tailored and flexible way), and significantly shaped the subsequent campaign. This learning might be useful for future public health campaign developers tasked with designing initiatives for similar target audiences.

**Conclusion:**

To promote brain health among younger populations, it is imperative to acknowledge their perspectives and needs. Targeted education was recognised as the first step towards raising awareness about the importance of maintaining brain health; this should address gaps in knowledge and the lack of accessible, accurate information. Approaching the topic of dementia, risk factors for neurodegenerative diseases and healthy lifestyle habits in a positive, tailored and flexible way were also perceived as key to influencing young people's motivation to engage in brain health promotional activities. These perspectives and preferences were reflected in the ‘Yo Me Celebro' campaign.

**Patient or Public Contribution:**

Four online and face‐to‐face meetings with a Young Adults Advisory Board of people aged 22 years were conducted. During these four meetings, young adults provided their perspectives on dementia prevention, dementia risk reduction and brain health promotion messages, and developed and reviewed the campaign materials.

## 1 Summary

Neurodegenerative diseases have complex etiologies and prognoses. Most of them are predominantly driven by a complex interplay between gene mutations, modifiable environmental influences and lifestyle factors, with modifiable factors accounting for at least 45% of the risk of developing dementia [1]. This has resulted in growing emphasis on strategies and interventions to maintain optimum brain health for as long as possible.

In this context, many associations and public health campaign developers have worked on initiatives targeting children and adults, aimed at informing, educating and raising awareness about dementia, brain health and healthy lifestyle habits. Some examples of these campaigns are ‘Move for your Brain’ Move for Your Brain – European Brain Council (EBC) by the European Brain Council, ‘My Amazing Brain’ My Amazing Brain | Brain Health by Alzheimer's Scotland, and ‘La salud cerebral’ Salud cerebral ‐ CEAFA by the Confederación Española de Alzheimer y otras demencias (CEAFA).

However, the voice of young adults is absent, despite being highly exposed to several modifiable risk factors and changes in health behaviours. To our knowledge, there are no campaigns specifically developed to inform, educate and promote brain health among adults in their early 20 s.

In this article, we provide an overview of the feedback collected from the Public Involvement consultations with young adults (i.e., people in their early 20 s) taking part in an exploration of young adults' perspectives of dementia and promoting messages on brain health. This feedback subsequently informed the design of the ‘Yo Me Celebro' campaign, developed as part of the ADIS (Early Diagnosis of Alzheimer's Disease by Immune Profiling of Cytotoxic Lymphocytes and Recording of Sleep Disturbances) project [2].

## 2 Introduction

### 2.1 Introduction

Dementia research has recently shifted its focus towards the development of diagnostic tools for earlier detection and risk prediction of dementia. There is also an increased attention on strategies to reduce modifiable risk factors that contribute to the onset of neurodegenerative diseases and their progression to maintain brain health for as long as possible. The ADIS project, which stands for ‘Early Diagnosis of Alzheimer's Disease by Immune Profiling of Cytotoxic Lymphocytes and Recording of Sleep Disturbances' is one of such projects with a focus on early diagnosis via the monitoring of two important risk factors (inflammation and sleep). ADIS is a collaborative effort between experts in data science, biotechnology, clinical research and Public Involvement (PI) to improve early diagnosis of Alzheimer's disease (AD) by combining an immune profiling of cytotoxic lymphocytes and recording of sleep disturbances. This research project ran for 3 years with a budget of 1.3 million EUR, until June 2025.

Emerging adulthood, which falls between 18 and 25 years of age, is a period characterised by transitional events, such as leaving home and moving to another city, starting university education, finding employment and taking on new responsibilities and risks. These transitional events towards adulthood modify exposure to environmental and lifestyle factors, and they particularly co‐occur or are increased at this age [3]. These transitional events come with growing independence, which is associated with increasing decision‐making responsibility. The confluence of these factors (changes in exposure to modifiable risk factors and decision‐making capacity), marks this period as an important phase, which may influence long‐term brain health. In their paper, Farina et al. note that to optimise brain health and well‐being in future generations, we must focus on empowering young adults to become active agents in the optimisation of their own brain health [4].

Biologically, emerging adulthood is also characterised by the fine‐tuning of the brain. The brain has already stopped growing in size by early adolescence. However, the young adult brain is highly malleable and adaptable, with brain architecture becoming more stable in the period between the late 20 s and the early 30 s [5, 6, 7]. During this period, the brain has a unique capacity to strengthen its resilience to future pathological changes [5, 6, 7]. At the same time, it is particularly sensitive to positive and negative experiences and other stressors, which can influence development [8]. This emphasises the profound impact that life experiences have on the development of the brain across the entire lifespan as they enhance personal development and increase the chances of developing adaptative competencies to navigate future stress, adversity or trauma that maintain psychological well‐being, a process known as resilience [9]. Early adulthood therefore seems to be a crucial period to target modifiable risk factors that influence brain aging in the long run. Therefore, this period arguably represents a key phase where brain health could be targeted through education and awareness campaigns.

Within the ADIS project, this transitional phase was identified as an important target through which risk factors such as inflammation and sleep hygiene could be optimised. Thus, it was decided to explore this topic further to determine whether it resonated with the target audience. As the intent was to inform the project, and to ensure people who are affected by the research have their ‘right to have a say' recognised, it was decided that this could be explored via a PI approach.

This report summarises feedback collected as part of PI consultations conducted by Alzheimer Europe (AE), a partner within the ADIS consortium, and describes how this subsequently informed the development of messages on brain health as part of a campaign to promote healthy lifestyle habits across the life course in young adults.

### 2.2 Background to the Paper

The concept of brain health first appeared in the literature in 1989 [10]. Although its use has increased substantially in the past 15 years, the concept remains surrounded by extensive debate. As a result, brain health has been conceptualised and interpreted in various ways, ranging from the ability to bounce back from adversity, the biological structure and function of the brain, or the absence of a neurological condition [10]. More recently, brain health has been more comprehensively defined by the World Health Organisation (WHO) as a 'state of brain functioning across cognitive, sensory, social‐emotional, behavioural and motor domains, allowing a person to realise their full potential over the life course, irrespective of the presence or absence of the disorders' (World Health Organization [11]). The significance of brain health in recent years has also been globally reinforced by the publication of the WHO position paper from 2022 [12]. In their position paper ‘Optimising brain health across the life course’, WHO emphasise that the state of brain health can fluctuate across all stages of life, and they advocate for the optimisation of brain health across the lifespan, not only because it reduces the prevalence of neurodegenerative and neurological disorders but because brain health influences physical health and overall well‐being.

De Rooij and Boots [13] note that, despite the extensive literature available, most brain health research focuses on older adults and addresses cardiovascular and neurodegenerative diseases. The authors argue that in the young, there are different development stages, and that in the majority of them, there are no cardiovascular, neurodegenerative or neurological disorders. Since brain health is not well explored in this population, it seemed imperative to understand how young adults conceptualise brain health, not only to frame the subsequent campaign but also to optimise brain health across generations.

### 2.3 Aim

The aim of this paper is to report on feedback collected from PI consultations conducted with young adults. Consultations were focused on (1) young adults' perspectives of dementia, and (2) messaging promoting brain health among people their age. Perspectives obtained from meetings were summarised and subsequently used to develop messages to promote brain health as part of the campaign ‘Yo Me Celebro' targeting young adults. The poster and videos of the campaign are available on AE's Instagram and TikTok channels Alzheimer Europe (https://www.instagram.com/alzheimereurope/https://www.tiktok.com/@alzheimereurope) • Instagram photos and videos/AlzheimerEurope (https://www.tiktok.com/@alzheimereurope) | TikTok).

## 3 Approach

### 3.1 Public Involvement in Dementia Research

The AE model for involving people with dementia in research has been explained elsewhere and is not reiterated here [14, 15]. In the field of dementia research, PI is the active involvement of people with dementia, people who are at risk of developing dementia, unpaid carers, and members of the general public with an interest in dementia in research projects, other than as research participants. This involvement typically means people work closely with researchers, sharing with them their perspectives, experiences and needs with regard to the research topic, and how the study is designed and conducted to ensure that activities are relevant, acceptable and meaningful to their intended audiences. Public Involvement differs from research participation and, also, from Public Engagement, which focuses on raising awareness, and disseminating information, knowledge and research findings to the wider public. The distinction between PI and qualitative research has also been a subject of discussion due to the boundaries between them often being blurred [16, 17]. While PI may employ methods and approaches similar to those used in qualitative research, the two differ fundamentally in their purpose. Qualitative research involves the collection of qualitative data, which is analysed in order to generate data to answer research questions, whereas PI is undertaken to inform and guide the development of research or other initiatives.

### 3.2 Design and People Involved

The PI work presented in this article was developed in the context of the ADIS project (it stands for ‘Early Diagnosis of Alzheimer's Disease by Immune Profiling of Cytotoxic Lymphocytes and Recording of Sleep Disturbances’). Although there were multiple PI strands within the ADIS project, this article reports the work conducted with the ADIS Young Adults Advisory Board (ADIS YA‐AB), which was composed of nine adults in their early 20 s (at the time of the consultations, they all were 22, seven women and two men). At the time of participation, the young adults involved were biomedical science students at the University of Barcelona (Barcelona, Spain) who were enroled in courses facilitated by one of the authors (D.B.‐F.). During seminars, the same author introduced the ADIS project and the idea to explore brain health with young adults. Those interested in contributing to the project were invited to put themselves forward as an ADIS YA‐AB member. The only eligibility criteria for involvement were interest in the topic and proficiency in Spanish, as the campaign materials were to be developed in this language.

Nine students agreed to join the ADIS YA‐AB. An online meeting was subsequently held, during which students received further information about the ADIS project, including the expected time commitment, the approximate number of online and face‐to‐face consultations, and AE's approach to PI.

Students were also asked about their motivations for joining the ADIS YA‐AB. Most of them expressed an interest in neurodegenerative diseases (for some of them, a personal experience was the motivating factor). They also voiced an interest in learning more about ways to promote health and well‐being. Others highlighted the value of participating in a collaborative project that would allow them to apply theoretical knowledge in a practical context.

Broader recruitment, designed to include young people with different academic/professional backgrounds was attempted by two authors (D.B.‐F. and S.M.‐B.). However, these attempts were not successful.

The core work for the PI activities with the ADIS YA‐AB was focused on collecting feedback (through video‐recordings and manual transcripts) on young adults' perspectives of dementia and potential messaging promoting brain health. This feedback informed the conceptualisation and development of the ‘Yo Me Celebro' campaign, which took place throughout 2024 and 2025. Three 2‐h online consultations were organised between March and July 2024 and one face‐to‐face consultation was organised in February 2025. For the first two online consultations, the group (*n* = 9) was divided into three small groups to facilitate the discussion and ensure time for contributions from all the members. One week before the consultations, members of the ADIS YA‐AB received the pre‐reading materials (these can be shared upon request and they are only available in Spanish), which contained background information and examples of the questions to be addressed during the consultations. These first two consultations focused on the following topics: 1. Knowledge and terminology surrounding brain health, dementia, neurodegenerative diseases, prevention and risk; 2. The importance of brain health; 3. Motivators, facilitators and barriers to adopting a healthy lifestyle; 4. The concept of successful ageing; 5. Lessons from older adults with optimal brain health; and 6. Stigma and Alzheimer's disease. Not all the feedback was directly relevant to the health promotion material subsequently developed (notably the topics exploring successful ageing, lessons from older adults and stigma). However, they also provided important context. The penultimate consultation, also held online, centred on developing content ideas based on the feedback received previously for the campaign poster and videos targeting young adults. The content was designed to reflect perspectives shared in earlier consultations. A final face‐to‐face consultation was aimed at reviewing and finalising the content.

All consultations were planned and facilitated by the first author S.M.‐B. Co‐authors D.B.‐F. and J.R. attended and co‐facilitated the online consultations, A.D.‐P. supported the planning and co‐facilitated the final face‐to‐face consultation. With permission from the members of the ADIS YA‐AB, video recordings were taken during consultations for the purpose of supporting manual transcription and summarisation of the consultations done by S.M.‐B. The same author drew upon some of the stages of thematic analysis to organise, summarise and present the feedback provided, that is, the feedback was examined for the recurrent or most important concepts raised by the members of the ADIS YA‐AB. These summaries were the basis of the health promotion messages in terms of content, ethos and style. Initial ideas were developed in a partnership approach and resulted in the ‘Yo Me Celebro' campaign.

Two additional consultations were held to gauge wider views of the content, initial insights into materials and how brain health was portrayed. First, the ADIS Advisory Board (ADIS‐AB), which was composed of older adults with mild cognitive impairment due to AD, reviewed the materials. Second, the advisory board of the Pattern‐Cog (Personalised ageing pattern for early risk detection and prevention of cognitive impairment and dementia in cognitively healthy individuals) project, an ERA‐PerMed‐funded project aimed at developing tools to predict the risk of cognitive impairment, provided an additional perspective on the potential applicability of the campaign across European countries.

## 4 Summary of the Feedback Provided and Description of Campaign Development

Personal understanding of brain health, terminology for the brain health campaign, barriers and facilitators to implementing healthy lifestyle habits when you are in your early 20 s, and the approach of this brain health campaign for young adults were the main topics of discussions, of which we provide more details below. The different categories of discussion have been illustrated with selected quotes provided by the members of the ADIS YA‐AB (more selected quotes are provided in Supporting Information: S1). Quotes (close paraphrases) are included to illustrate the type of feedback received. They should not be related with qualitative research methods which typically also reproduce quotes in support of a theme. Rather, they are presented to demonstrate to the reader the type of feedback received and how it then shaped the collaboratively produced campaign materials.

### 4.1 Personal Understanding of Brain Health

When asked about a future brain health education campaign in early consultations, the ADIS YA‐AB highlighted the lack of uniformity in how the term ‘brain health' is interpreted and understood. Some young adults conceptualised brain health as the absence of symptoms or just to the absence of brain damage, whereas for others, brain health is someone's ability to function properly in different domains. This lack of a common understanding of ‘brain health' makes difficult not only to define the concept itself, but also to determine how brain health can be promoted and maintained from an early age and throughout life, even in the absence of cognitive problems. At the project level, this underscored the importance of clearly stating how to frame and promote a healthier brain across the lifespan.

“When that change [damage] interferes with your ability to carry out certain activities of daily living, then you no longer have brain health. But if that change at the brain level does not interfere with activities of daily living, then that person has brain health.”

### 4.2 Terminology for the Brain Health Campaign

To understand how to frame and promote a healthier brain across the lifespan, we explored three potential expressions with the members of the ADIS YA‐AB that could be adopted in health promotion materials: (1) dementia prevention, (2) dementia risk reduction, and (3) brain health care. Members were asked about their opinions, preferences and concerns in relation to each expression.

Members associated ‘dementia prevention' with something that is 100% preventable, and thus potentially inaccurate. There were concerns that this terminology could result in misconceptions about control and personal responsibility (potential self‐blame). The expression ‘dementia risk reduction', was interpreted as something someone can do to reduce their chances of developing dementia. That is, you can do *A* to reduce the chances of *B* happening to you, but *B* can still happen to you. The expression may, in their opinion, raise awareness of the preventability of neurodegenerative diseases. However, the word ‘dementia' was perceived as potentially problematic by young people as dementia is usually seen as something that happens to older people, that is, they will hopefully be old one day but old age seems so far off. Finally, the expression ‘brain health care' was perceived as the most positive and accessible terminology that encouraged, emphasised and normalised brain protective behaviours. Furthermore, this expression was closer to the concept of self‐care, which young people related to and which has a focus on behaviours that could be adopted now rather than on a condition perceived as distant and primarily affecting older adults.

This discussion exemplified the importance of the terminology in any materials presented to younger audiences and was key in guiding both terminology (brain health care) and ethos (positivity). It also indicated a strong direction for the campaign, that is, imparting practical self‐care style advice to support brain health and well‐being across the lifespan.

“When you talk about care, I understand it as something I can do myself for my brain, and that is good, because there are so many people who don't know that the brain can be taken care of. The brain is an organ, it is part of us and it can also be taken care of.”

### 4.3 Barriers and Facilitators to Implementing Healthy Lifestyle Habits in Young Adults

During the consultations, barriers and facilitators that influenced the adoption of healthy lifestyle habits were discussed. Although members of the ADIS YA‐AB were aware of modifiable dementia risk factors, some struggled to link healthy lifestyle habits with brain function and health. Lack of information, misinformation, or unrealistic advice, the absence of tangible short‐term benefits, and time and motivation factors were important barriers that prevented the adoption or routinisation of habits that promote brain health. In these discussions, it was again noted that education in the form of useful and simple tips, could provide motivation. Their feedback also emphasised the need to create realistic, practical and affordable recommendations that could be easily interpreted and adopted within existing routines and circumstances.

“In my opinion, one of the aspects that contributes most to this laziness is the lack of knowledge about how to create and implement a routine that is suitable for me. I believe that providing instructions or ‘tips', instead of big and general recommendations (such as ‘exercise three times per week'), would motivate people more to adopt these habits.”

### 4.4 Campaign Slogan, Ethos and Content

Members of the ADIS YA‐AB stressed the importance of framing dementia in a positive and empowering way. They argued that emphasising the increased independency they experience at their age and the control they have over their own actions, rather than relying on restrictive or prescriptive messages, was important. This approach may work better for this target audience, who often view neurodegenerative diseases as conditions that are unlikely to affect them anytime soon. Reflecting this perspective, the ADIS YA‐AB suggested the campaign adopt the slogan ‘Yo Me Celebro' (‘I celebrate myself') and the group selected five preventative targets that linked to the concepts of positivity, increasing independency, control and self‐care. These targets were: sleep, social isolation, and infrequent social contact, sedentary lifestyle, diet, and mental and emotional health.

“I think we should focus on giving a positive message instead of trying to impose something. With impositions, it seems that they want to force us to deprive ourselves of something. And this is not going to work. So, the ‘stop doing this or that', as in stop smoking or stop drinking alcohol because it is bad for your brain is not going to work. A positive message can lead us to the fact that we should give up certain things that are not good for us.”

### 4.5 Summary of the Campaign

The feedback from the PI consultations presented above was used to inform the ‘Yo Me Celebro' campaign. This result—the campaign—consisted of a poster and a series of videos that were shared via AE's established networks including TikTok and Instagram accounts, using the hashtag #YoMeCelebro, which are two of the most used social media platforms among the younger demographic [18]. The poster was a campaign advertisement that visualised activities that may influence long‐term brain health. The first video, which was used to launch the campaign, featured the first author (i.e., S.M.‐B.) and concluded with an open‐ended question inviting the audience to reflect on what it means ‘celebrarse' (‘self‐celebration'). Subsequent videos expanded on this concept by exploring how self‐celebration can be expressed through different lifestyle behaviours and by providing practical examples of how young adults can incorporate these habits into their daily lives. These videos (*n* = 5) featured the young adults involved in the PI consultations. In their videos, they talked about the five modifiable risk factors for dementia that they believe most affect people their age (i.e., sleep, social isolation and infrequent social contact, sedentary lifestyle, diet, and mental and emotional health), how these can be shaped or influenced by positive self‐care routines and how they would practically achieve this.

## 5 Discussion of Findings

In concrete terms, the reflections acted as an overarching framework that shaped the approach and the content of the campaign. Young people voiced a preference for framing brain health in a positive light (i.e., not preventing dementia). They described being more attracted to positivity or self‐care messages (i.e., promoting/preserving) and dismissive of messages that they do not perceive to affect them (i.e., dementia is a disorder of the elderly and thus not applicable to them). Harnessing approaches or leveraging techniques developed in the wellness industry might be applicable to educational resources targeting this audience, as they were perceived as more appealing. Of equal importance was the need for accessible messaging, particularly given differences in education, experience and exposure to neurodegenerative diseases. At the same time as being accessible, information presented must also avoid being patronising or adopting a ‘top‐down, dictatorial approach', as this should reflect their growing independency and decision‐making responsibility. Similarly, topics that reflected their priorities as maturing, independent adults, and which they perceived to be within their control, should be foregrounded. These preferences were acknowledged and incorporated in the development of the ethos of the campaign slogan, and in the style and content of the associated video campaigns.

In addition to framing the topic in an appealing way and communicating it in language that is attractive to young people, members of the ADIS YA‐AB highlighted that we should consider how the messages can be delivered flexibly or in a tailored manner, to enable individual adoption. A flexible approach would also enable young people to adapt educational and practical messages in line with their growing independence and new experiences. Consequently, the videos adopted a personal narrative style with tips and tricks for change.

### 5.1 Comparison With Other Literature

We could not find direct comparisons in the PI literature. However, one expert opinion piece and one qualitative study offer relevant comparisons. Booi et al. conducted focus groups with 39 young adults in North America and Europe, to explore awareness and understanding of brain health and dementia risk factors. Similar to the reflections presented here, they describe how awareness and understanding vary and were influenced by multiple factors (including personal or familial experience). They similarly report how participants connected/did not connect lifestyle factors to brain health and dementia risk [19]. In a conceptual review and position paper, Farina et al., have proposed the concept of ‘Brain Capital' as a way of targeting brain health messages at young adults. Similarly to our summary, they emphasise the importance of a positive brain health movement, and they call for the involvement of young people to shape this movement [4].

### 5.2 Limitations

Whilst we present some important findings that may be relevant to future initiatives, there are some limitations that must be considered:

• The feedback collected during the discussions with the members of the ADIS YA‐AB (all are from Spain) informed the ‘Yo Me Celebro' campaign, which targeted Spanish‐speaking young adults. We believe it would have been very helpful to involve young adults from other countries in the discussions. This would have helped us understand national nuances and preferences and ensure messages are adapted to different languages and cultures.
• All of the members of the ADIS YA‐AB were university students enroled in biomedical‐related courses. All of them had some knowledge of dementia, neurodegenerative diseases, lifestyle habits, and brain health. We did not manage, however, to involve any young adults enroled in a different career path or with a different educational background (e.g., students enroled in non‐biomedical courses or young adults who did not enrol in any university degree). Research has previously shown that knowledge about dementia and dementia risk reduction is very limited, even among people indirectly affected by dementia [20]. Therefore, involving young adults with different backgrounds and levels of education would have allowed us to develop messages adapted to people with little or no knowledge of brain health, dementia and healthy lifestyle habits.

### 5.3 Future Efforts

The work presented in this article was conceptualised and conducted within the framework of PI and not qualitative research. We, the authors, emphasise that methods used in PI sometimes resemble those used in qualitative research (e.g., focus groups). However, the use of specific methods does not determine whether something is PI or research. Rather, it is the reason for using a particular method and how the information is used that are the determining factors (i.e., if answering a research question, then it is research. If informing a study or providing a voice, then it is PI [21]). Moreover, PI involves addressing a topic together with researchers, not providing them with data that is subsequently analysed. The input and reflections from the members of the ADIS YA‐AB contributed to the development of messages on brain health to promote healthy lifestyle habits among young adults, and had an impact on the subsequent development of the poster and videos of the ‘Yo Me Celebro' campaign.

The authors felt that summarising and publishing the rich perspectives shared by the members of the ADIS YA‐AB could be useful for researchers or public health campaign developers working on similar initiatives or campaigns for similar target audiences. We hope that this paper will promote PI work and highlight the value of involving members of the public in research and/or development of public health campaigns other than as research participants.

It is important to note that the work presented in this paper, and the input and reflections provided by the members of the ADIS YA‐AB, were not collected for research purposes or within the framework of qualitative research. Nevertheless, this work could be refined further and lay the foundations for future qualitative research.

## 6 Conclusions

Dementia is a complex societal problem that touches us all, regardless of age and familiarity with neurodegenerative diseases. The first step to tackle this problem is to acknowledge the brain as a part of our body that can be taken care of and must be maintained, not from the moment we are at risk of developing a neurodegenerative disease, but from the moment we have the ability to take responsibility for it. Education and information are therefore key, particularly during emerging adulthood, when young adults are particularly exposed to various transitional events and new life experiences. The way information is given and how we frame the topic are also key. Unrealistic and non‐tailored recommendations and difficult‐to‐implement lifestyle habits may not work for young adults. Instead, a positive and flexible approach that gives value to activities that, in many cases, are already part of young adults' daily lives (e.g., meeting friends, exercising outdoors) may support positive behaviour changes with potential longer term impact.

Besides extolling the benefits that simple activities have for our brain health; the slogan of this campaign, ‘Yo Me Celebro', helps us remember that our body is our lifelong home and our most precious asset, and that we can do a lot to celebrate (i.e., take care of) it.

## Author Contributions

**Soraya Moradi‐Bachiller:** conceptualisation, writing – original draft, methodology, writing – review and editing. **David Bartrés‐Faz:** writing – review and editing. **Holger Fröhlich:** writing – review and editing. **Jean Georges:** writing – review and editing. **Dianne Gove:** supervision, writing – review and editing. **Jesús Rodrigo:** writing – review and editing. **Jussi Tohka:** writing – review and editing. **Ana Díaz‐Ponce:** writing – review and editing, supervision.

## Conflicts of Interest

The authors declare no conflicts of interest.

## Supporting information


Supporting File

## Data Availability

The authors have nothing to report.
