# Supplementary material for: Young People's Voices in Developing Messages on Brain Health: An Initiative to Promote Brain‐Protective Lifestyle Habits Among Young Adults
Source: Health Expect. 2026 Oct 25;29(5):e70926. doi: 10.1111/hex.70926 (PMC13696039; doi:10.1111/hex.70926)
Supplement: Supplementary file 1 [file HEX-29-e70926-s001.docx]

**Supporting File 1**

**Personal understanding of brain health**

"I understand brain health as the absence also of brain pathology, that the neural structures function well, so that all the functions derived from them work properly.”

"When I think of brain health, I understand it more as damage at the physiological level that does not necessarily translate into something visible. For example, there can be neuronal death without the manifestation of any symptoms.”

“It's true that health is the absence of pathology. And pathology, clinically, is defined as the presence of symptoms. If you don't have symptoms, then I would say that the person is healthy, even if they have the biomarker. The biomarker would only be a tool for early diagnosis, a way of anticipating that the person will have the disease.”

“There is always the pathological component; anatomically, there is an alteration, but the fact that it does not manifest itself means that the person has brain health in that sense. Clinically, until it manifests, you can say that they have brain health, but biologically, if there is an alteration, then there is no health. It depends on how you look at it.”

**Terminology for the brain health campaign**

“I don't believe that dementia can be prevented, but I do believe that the risk of developing dementia can be reduced. I don't think you can refer to prevention of dementia as action (i.e. adopt a healthy lifestyle) = consequence (i.e. no risk at all).”

“I see prevention more as if you do this, it won't happen to you. And risk reduction is reducing the likelihood, but it can still happen to you.”

“I think that with risk reduction, you are putting on the table that we all start from a baseline risk that we can somehow control with our actions (reduce it or let it increase). It puts you on notice. It tells you that you also have the control to modify it with changes in your lifestyle habits. The expression ‘dementia prevention’ kind of takes away the factor ‘you can have it [the disease] too, regardless of what you do’.”

“I think that with the word prevention, you are assuming that you go to a 0% risk when you change certain things in your lifestyle, and with reduction, you don’t go to 0%. In other words, with the word prevention, you are saying that there will be no risk at all if you adopt a healthy lifestyle.”

“If I see the expression ‘dementia prevention’ in a campaign, I would feel disappointed, because if you say prevention, you create expectations... and if I got the disease in the future, I would think, why did I get it when I did everything that had to be done?”

“If they [healthcare professionals] talk to you about prevention, they create expectations... and I would think ‘why me if I did everything I had to do?’”

“Among these three expressions [‘dementia prevention’, ‘dementia risk reduction’ and ‘brain health care’], I would choose the expression ‘brain health care’ for the campaign because everything about dementia prevention or risk reduction is, in the end, very far away. Brain health care is like more now, as in something you can do now.”

“Given the perspective of young people who may not have studied scientific or biomedical degrees, an expression such as ‘brain health care’ is more likely to resonate and sends a positive message too. The expression ‘dementia prevention’ is not entirely correct, so I would not use it.”

“I would choose the expression ‘brain health care’ for the campaign because the last generations have prioritised exactly this, i.e. self-care, and I think we are all more familiar with this expression than with the other two (i.e. ‘dementia prevention’ and ‘risk reduction’). I think if I read ‘dementia prevention’, I would think that it is a campaign for older people, but not for people my age.”

“The word dementia is something you associate with old age. The expression ‘brain health care’ includes everyone. The word dementia in a campaign for young people… I would mention it somewhere but I would focus more on brain health care as such. I would not use dementia prevention as the slogan or main message of the campaign.”

**Barriers and facilitators to implementing healthy lifestyle habits in young adults**

“It is hard to think that what you do now will have an impact on what will happen to you in 30 years. It's not that young people don't want to make a change; it is more that they don't know what the consequences of (not) doing something are. It's more misinformation and the lack of information than anything else. When you are young, maybe the last thing you care about is this [brain health] because you are not seeing what it will be useful for at the moment. What I mean is that you are not going to develop dementia right now. If dementia happens, it will happen in 40 years, so why should I care? Also, sometimes you don't know what to do [to take care of your brain] or you don't have the means to do it.”

“For example, for me, my problem with physical activity is laziness. When I have tried to do physical exercise 2 or 3 times a week, I’ve ended up giving up because of laziness and the university, which leaves us no time for anything else.”

“Before I went to university, I would have never thought of the brain as one of the organs that could benefit from healthy habits. I didn't think of the brain as a part of my body I have to and can take care of.”

“I don't think I had ever associated any healthy activity with the fact of taking care of the brain. I have heard of doing crosswords to exercise your brain, but I have never associated diet or exercise with brain health.”

“I think there is an important bias between people who study science and those who don't. I am from a small village. None of my friends from my village are studying anything related to science. When I started university [a scientific degree], I made friends who were studying science too, and that's when I changed my habits for the better. However, all my friends in the village still smoke, drink a lot... But I don’t think it is because they don’t want to change. The problem is that they don’t know what I know. If the message is communicated in the right way to reach people our age, now is a good time, because our generation has fully embraced the culture of self-care.”

“In my case, by associating the habit with an immediate reward, that healthy habit becomes less tedious to do and I overcome that initial laziness. This could be adapted to exercise, for example, listening to music or podcasts [reward] at the same time that you exercise. The additional entertainment of watching a video or listening to something while exercising has been very effective in my case.”

“I think our age… it's a time when we have lots of things to do, we want to try lots of things and you don't have much time. I think that, sometimes, the recommendations [to adopt healthy lifestyle habits] are a bit complicated to implement. Telling a young person, who is studying at the university, that they have to sleep 8 hours is not realistic, not because they don't want to, but because they don't have those hours a day to sleep.”

“Having a healthy diet is not a realistic recommendation for people our age. I think that healthy food is very expensive, things you can't afford [referring to bio things]. Or cooking too, because maybe you don't have time to do it.”

“In my case, I have learned what I have to eat to get all the nutrients I need. However, in the beginning, I was spending too much money in organic food. It is difficult because in the end you are going to live on your own and you have to learn how to buy groceries all by yourself.”

“I don't think ‘exercise’ is a feasible recommendation. Before I went to university, I never practiced any sports. When I started university, I wanted to join a gym. Firstly, it was very expensive and secondly, I was studying all day, I didn't have the time. So, I don't think it is feasible.”

“Stress is something very important that we identify quite well. What I think is that we don't have many ways of dealing with it or maybe we don’t know what we could do to deal with it.”

“Young adults can adapt to changes easily. I think that, at our age, one is still looking for new things to try, new experiences… and I think that it is at this age that you are more susceptible to adopting lifestyle changes. However, this can only work if you tell these young adults why and how they should make these changes in their lives. The why and how are very important.”

“What would I tell people to motivate them to have healthy lifestyle habits? I would tell them that they would feel much better and feel more energized to do things. And if they can do these activities with people they get along with, all the better, because they will enjoy themselves more. Taking care of yourself makes you feel good.”

“What motivates me is the fact that I make time for myself. We have lots of things to do and taking care of myself is just time for me. It is like I'm doing it just for me.”

“I think the motivation to adopt healthy habits can increase with knowledge. In other words, discovering the beneficial effects of a habit can motivate the person to make it part of their daily routine. So, the simple fact of informing about the beneficial effects would already be helping to raise awareness.”

**Campaign slogan, ethos and content**

“I think that the expression ‘now that I can’ gives a kind of positive reinforcement to the message. You are telling the person that they have the power, they have the control, that now they have more independence to decide on so many aspects of their lives. You are not forcing them to change their habits, you are simply telling them that they have the power to do it. It is a subliminal way of encouraging people without forcing them.”

“I think that being able to make decisions on our own, that independence, is positive and is the motivation to change. When they stop imposing what we have to do, we start thinking of what is best for us, and what we really want for our lives.”
